# Hierarchical Molecular Events Driven by Oocyte-Specific Factors Lead to Rapid and Extensive Reprogramming

**DOI:** 10.1016/j.molcel.2014.06.024

**Published:** 2014-08-21

**Authors:** Jerome Jullien, Kei Miyamoto, Vincent Pasque, George E. Allen, Charles R. Bradshaw, Nigel J. Garrett, Richard P. Halley-Stott, Hiroshi Kimura, Keita Ohsumi, John B. Gurdon

**Affiliations:** 1 Wellcome Trust/Cancer Research UK Gurdon Institute, Tennis Court Road, Cambridge CB2 1QN, UK; 2 Department of Zoology, University of Cambridge, Cambridge CB2 1QN, UK; 3 Graduate School of Frontier Biosciences, Osaka University, Suita 565-0871, Japan; 4 Laboratory of Molecular Genetics, Division of Biological Science, Graduate School of Science, Nagoya University, Furo-cho, Chikusa-ku, Nagoya, Aichi 464-8602, Japan

## Abstract

Nuclear transfer to oocytes is an efficient way to transcriptionally reprogram somatic nuclei, but its mechanisms remain unclear. Here, we identify a sequence of molecular events that leads to rapid transcriptional reprogramming of somatic nuclei after transplantation to *Xenopus* oocytes. RNA-seq analyses reveal that reprogramming by oocytes results in a selective switch in transcription toward an oocyte rather than pluripotent type, without requiring new protein synthesis. Time-course analyses at the single-nucleus level show that transcriptional reprogramming is induced in most transplanted nuclei in a highly hierarchical manner. We demonstrate that an extensive exchange of somatic- for oocyte-specific factors mediates reprogramming and leads to robust oocyte RNA polymerase II binding and phosphorylation on transplanted chromatin. Moreover, genome-wide binding of oocyte-specific linker histone B4 supports its role in transcriptional reprogramming. Thus, our study reveals the rapid, abundant, and stepwise loading of oocyte-specific factors onto somatic chromatin as important determinants for successful reprogramming.

## Highlights

• *Xenopus* oocytes induce an oocyte transcription pattern in mouse nuclei in 2 days
• Reprogramming requires a switch from somatic to oocyte transcriptional components
• Unusually high amounts of oocyte-derived RNA polymerase II drive reprogramming
• The pattern of oocyte linker histone binding to somatic chromatin is revealed

## Graphical Abstract

**Figure:**
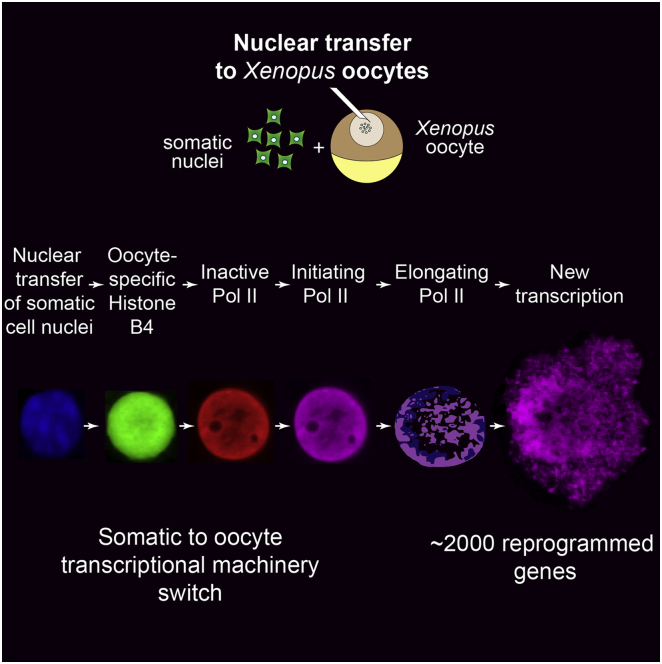

## Teaser

Jullien et al. identify a sequence of events leading to rapid transcriptional reprogramming of somatic nuclei after their transplantation to *Xenopus* oocytes. Transcription is switched selectively to an oocyte-type program in a process involving stepwise loading of oocyte-specific factors onto transplanted chromatin.

## Introduction

Nuclear reprogramming is of much current interest, especially in view of the potential therapeutic value of cells reprogrammed directly from patients (Tachibana et al., 2013, Wu and Hochedlinger, 2011). However, very little is at present known about the mechanisms of nuclear reprogramming (Narbonne et al., 2012, Plath and Lowry, 2011, Wu and Hochedlinger, 2011). An understanding of the mechanisms required to induce and maintain cell identity is crucial to improve the efficiency, quality, and safety of reprogrammed cells and largely relies on our ability to understand mechanisms of gene regulation during reprogramming. While much interest resides in reprogramming to induced pluripotent stem cells (iPSCs), other routes toward reprogramming, such as nuclear transfer (NT) and cell fusion, provide unique experimental advantages to dissect the steps and mechanisms of transcriptional reprogramming, even without the need for cell division in some experimental settings. Furthermore, the transfer of nuclei to second meiotic metaphase oocytes can result in reprogrammed pluripotent cells of high quality and with high efficiency (Kim et al., 2010, Le et al., 2014, Tachibana et al., 2013). For *Xenopus* first meiotic prophase oocyte NT experiments, several hundred mouse somatic cell nuclei are injected into the specialized oocyte nucleus (the germinal vesicle; GV), leading to changes in transcription of the incoming somatic nuclei within a few days in the absence of cell division (Halley-Stott et al., 2010) (hereinafter, oocytes refer to cells in first meiotic prophase). It was previously demonstrated that the oocyte system is a useful tool to reveal important factors for the establishment or maintenance of cell identity, which are directly applicable to several other reprogramming systems, such as mouse and human iPSC and mouse NT (Wen et al., 2014, Barrero et al., 2013, Gaspar-Maia et al., 2013, Miyamoto et al., 2013, Pasque et al., 2011, Pasque et al., 2012).

To further understand reprogramming by oocytes, transcriptional analysis of individual genes has been used at different time points after NT of mouse somatic nuclei (Byrne et al., 2003, Halley-Stott et al., 2010). For example, we previously showed that the pluripotency gene *Sox2*, as well as lineage-specific gene *MyoD*, are reactivated after NT (Biddle et al., 2009, Jullien et al., 2010). Normal fully grown oocytes actively transcribe repetitive and single-copy genes and are characterized by the formation of actively transcribing chromosomes, called lampbrush chromosomes (Callan, 1986, Gall and Murphy, 1998, Gall, 1954). The rate of an oocyte’s own transcription is extremely high and has been estimated to be a thousand times greater than that of a somatic cell (Davidson, 1986). Given these data, a model has emerged in which numerous genes are active after oocyte somatic cell NT to conform to a lampbrush-type transcription in which almost all genes are transcribed (Simeoni et al., 2012). However, because many transcripts from the incoming somatic nuclei are carried over during the NT procedure, it has not been possible to comprehensively analyze the transcriptome of reprogrammed nuclei.

Mechanistically, oocyte-enriched factors, such as the oocyte linker histone B4 (B4), have been implicated in the reactivation of a few pluripotency genes such as *Sox2* (Jullien et al., 2010), but their genome-wide and gene specific requirements are not known. Moreover, there have been few insights into the temporal sequence of molecular events that drive the reprogramming process. *Xenopus* oocytes contain enough RPB1, the catalytic subunit of RNA polymerase II (Pol II), for the transcription of 10,000 somatic nuclei, yet only a very small fraction of RPB1 is phosphorylated and actively transcribing the oocyte lampbrush chromosomes (Bellier et al., 1997, Doyle et al., 2002, Roeder, 1974).

To understand the changes leading to the reprogramming of somatic nuclei by NT to the oocyte, we have used time-course analyses at the single-nucleus level, defining different steps of reprogramming and demonstrating that the somatic transcriptional machinery is exchanged for that of an oocyte in a hierarchical manner, which does not require new protein synthesis, and leads to a greatly increased level of Pol II binding and phosphorylation in transplanted nuclei. Using genome-scale gene expression analysis to specifically profile newly synthesized transcripts from transplanted somatic nuclei, we demonstrate that oocytes induce extensive, rapid, and specific transcriptional patterns distinct from the somatic type. We further demonstrate by chromatin immunoprecipitation sequencing (ChIP-seq) analyses that the binding of oocyte linker histone B4 contributes to transcriptional reprogramming in transplanted nuclei.

## Results

### Direct Genome-wide Transcriptional Reprogramming within 48 hr following Nuclear Transplantation to Oocytes

**Figure 1:**
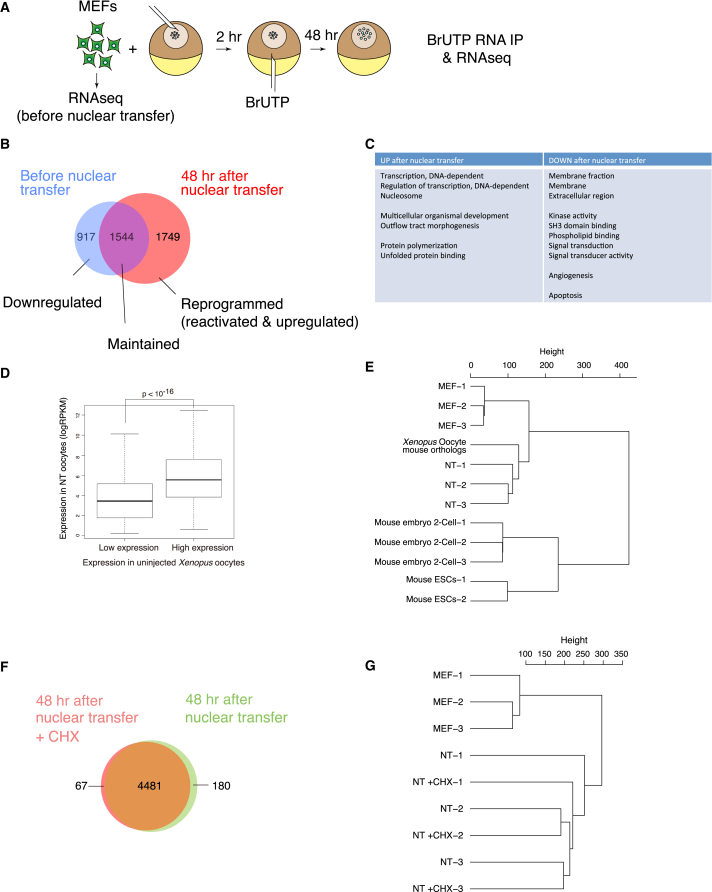
Rapid Genome-wide Transcriptional Reprogramming in the Absence of Protein Synthesis (A) Experimental design for the RNA-seq analysis of newly transcribed mRNAs before and after NT to *Xenopus* oocytes. BrUTP is used to label newly transcribed RNAs. (B) Venn diagram of genes classified as activated (reprogrammed), continuously transcribed (maintained), or repressed (downregulated) after NT based on log_2_ count per million (log_2_ CPM donor cell divided by NT). False discovery rate (FDR) < 0.05. Results are based on three independent experiments. (C) Representative examples of GO terms significantly enriched (FDR < 0.05) in genes upregulated (reprogrammed genes in Figure 1B) and downregulated (downregulated genes in Figure 1B) after nuclear transfer. (D) Highly expressed genes in *Xenopus* oocytes tend to be highly expressed in transplanted nuclei. Box plots of gene expression data, with boxes demarcating the 25th–75th percentile and the median indicated by black lines. Whiskers extend to the most extreme data points with a distance to the box at most 1.5 times the box height. Expression levels are compared by reads per kilobase per million (RPKM) values. Statistical significance was calculated using t test; p < 10^−16^. (E) Hierarchical clustering of gene expression from cultured MEFs before NT, mouse orthologs of *Xenopus* oocytes expressed genes, MEFs after NT to oocytes, mouse ESCs, and two-cell-stage mouse embryos. (F) Differentially expressed genes after NT in the presence or absence of CHX treatment. Genes are considered differentially expressed if log_2_CPM (control/CHX treatment) shows a FDR value < 0.05. Results are based on three independent experiments. (G) Hierarchical clustering of gene expression from cultured cells before NT, NT oocytes, and NT oocytes with CHX treatment. See also Figure S1 and Tables S1 and S2.

To define the molecular basis of transcriptional reprogramming by *Xenopus* oocytes, we determined how the transcriptome of mouse somatic cells changes after NT into the germinal vesicle of oocytes. Specifically, we compared the polyA+ messenger RNAs (mRNAs) accumulated in cultured immortalized mouse embryonic fibroblasts (MEFs) to those produced during the 2 days after transplantation of MEF nuclei to *Xenopus* oocytes. Transcripts produced exclusively after NT were selectively labeled using 5-bromouridine 5′-triphosphate (BrUTP), which we injected into oocytes, allowing newly transcribed mRNAs to be immunoprecipitated before the generation of RNA-sequencing (RNA-seq) libraries using a protocol adapted for low cell number (Core and Lis, 2008, Tang et al., 2010) (Figure 1A).

RNA-seq analysis revealed that NT of mouse nuclei to *Xenopus* oocytes induces a rapid, genome-wide shift in transcription (Figure 1B; Table S1 available online). We defined genes as expressed only when transcripts were detected in all triplicate samples (counts per million > 1), assuring that genes identified in this analysis are reproducibly expressed. Using this stringent parameter, we identified 4,210 genes as expressed in donor cells, in NT oocytes or in both. Forty-eight hours after NT, 917 genes (21.8%) were downregulated (Figure 1B, downregulated), while 1,544 genes (36.7%) expressed in donor nuclei remained expressed after NT (Figure 1B, maintained). It is important to note that activated and upregulated mouse genes after NT accounted for more than 40% of mouse genes expressed in NT oocytes (Figure 1B, reprogrammed, 1,749 genes).

Gene ontology (GO) analysis showed that reprogrammed genes after NT include GO categories relevant to oocyte functions, such as those for transcription and development, while downregulated genes are mostly enriched in signaling pathways (Figure 1C), which might reflect loss of somatic cell properties. We investigated further the possibility that MEF nuclei were reprogrammed to an oocyte type of transcription. We specifically asked whether mouse orthologs of *Xenopus* genes highly expressed in oocytes are preferentially expressed in MEF nuclei after NT. To this end, we performed RNA-seq analysis of untransplanted *Xenopus* oocytes (Table S2) and defined two gene expression sets based on low and high expression, respectively (Figure 1D). In mouse nuclei transplanted to oocytes, mouse orthologs of genes highly expressed in *Xenopus* oocytes showed clear preferential expression over orthologs expressed at low levels in oocytes (Figure 1D, p < 10^−16^). These results suggest that NT to *Xenopus* oocytes induces a rapid, extensive shift in transcription from a somatic- to oocyte-type.

To characterize this shift further, we asked whether the pattern induced in mouse somatic nuclei after NT to the *Xenopus* oocyte differs from a mouse embryonic stem cell (ESC) transcriptional pattern. We performed a hierarchical clustering of gene expression level in mouse ESCs, two-cell-stage mouse embryos, *Xenopus* oocytes (mouse orthologs) and in MEFs before and after NT. ESCs and two-cell-stage embryos clustered away from other samples, while NT samples clustered together with *Xenopus* oocyte mouse orthologs, away from MEFs before NT (Figure 1E). This rapid genome-wide reprogramming implies that the oocyte is endowed with a robust and abundant transcriptional machinery able to rapidly and specifically reprogram hundreds of somatic nuclei to an oocyte pattern, distinct from a mouse pluripotent stem cell pattern.

Unlike other reprogramming systems, the transcriptional reprogramming observed here takes place without ongoing DNA synthesis (Gurdon, 1968). We next asked whether this reprogramming relies only on components accumulated in the oocyte or whether it also requires proteins synthesized after NT. We inhibited translation during reprogramming using cycloheximide (CHX; Figure S1). Notably, rapid genome-wide changes in transcription took place regardless of translation inhibition; the latter did not prevent reprogramming by the oocyte (Figure 1F). Indeed, the effect of CHX treatment was limited to the activation of a small additional set of genes (<5%). To further test the effect of CHX on genome-wide transcriptional reprogramming by oocytes, we performed hierarchical clustering of the gene expression level before and after NT and with or without CHX. Untransplanted MEF samples clustered together, away from NT samples, all of which clustered together regardless of CHX addition (Figure 1G). Thus, inhibition of protein synthesis does not prevent transcriptional reprogramming by the oocyte. We conclude that protein synthesis is dispensable for reprogramming by *Xenopus* oocytes, suggesting that all of the factors required for reprogramming are present at the time of NT. Therefore, following NT, the maternal components stored in the oocyte trigger a genome-wide shift in transcription.

### Nuclear Reprogramming by Oocytes Is Hierarchical and Uniform over Time and within a Nuclear Population

**Figure 2:**
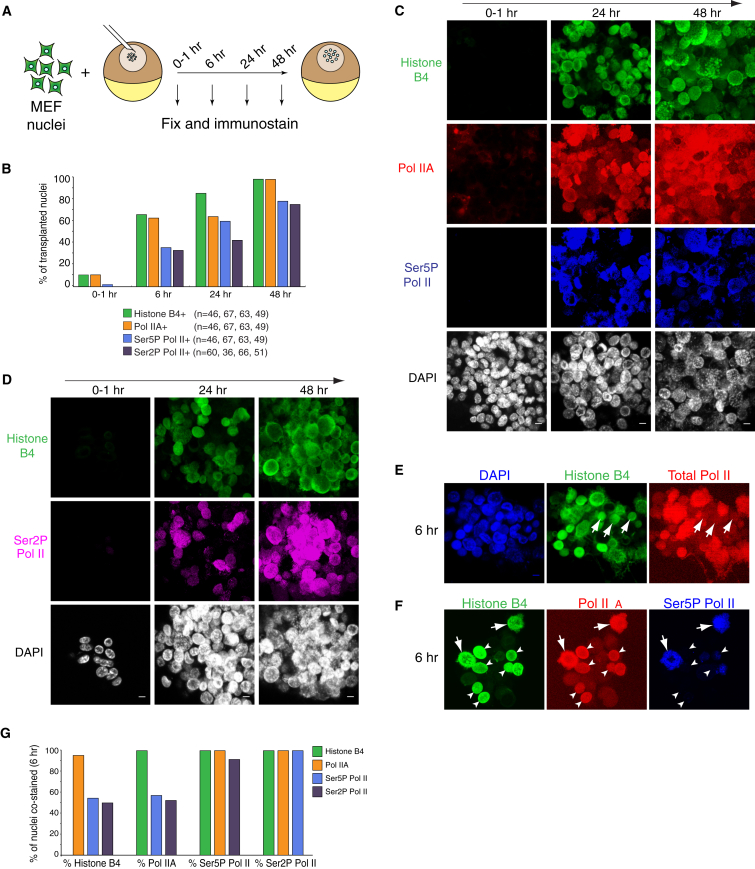
Time-Course Analysis at the Single-Nucleus Level Reveals the Temporal Recruitment of Oocyte B4 and of Pol II Phosphorylation (A) Experimental set-up to examine binding of oocyte factors to transplanted nuclei. (B) Proportion of transplanted nuclei stained by B4, hypophosphorylated Pol IIA, Ser5P Pol II, and Ser2P Pol II at different times after NT. n = number of nuclei scored. (C and D) B4 (green), Pol IIA (red), Ser5P Pol II (blue), and Ser2P Pol II (magenta) immunofluorescence detection at the indicated times after NT of MEF nuclei. DAPI is in white. Scale bars in DAPI, 10 μm. Images represent projected Z sections (C–F). (E) Immunofluorescence detection of B4 (green) and total Pol II (red) 6 hr after nuclear transfer of MEF nuclei. Total Pol II antibody recognizes hypophosphorylated Pol IIA, Ser5P Pol II and Ser2P Pol II. Arrows indicate nuclei that are positive for B4 but negative for total Pol II. DAPI is in blue. (F) Immunofluorescence detection of B4 (green), Pol IIA (red), and Ser5P Pol II (blue). Arrowheads indicate nuclei that are positive for B4 and Pol IIA but negative for Ser5P Pol II. Arrows show nuclei positive for all markers. (G) Proportion of transplanted nuclei stained with B4, Pol IIA, Ser5P Pol II, or Ser2P Pol II, which are also stained with B4 (green), Pol IIA (orange), Ser5P Pol II (blue), or Ser2P Pol II (magenta) at 6 hr. See also Figure S2 and Table S3.

RNA-seq analyses with CHX indicate that reprogramming is driven by factors already present in *Xenopus* oocytes. We therefore hypothesized that reprogramming is mediated by oocyte components that are abundantly present in the GV, since hundreds of somatic nuclei can be quickly reprogrammed. We focused on two abundant oocyte factors, histone B4 (Jullien et al., 2010) and Pol II. These are the starting point and endpoint of the reprogramming process. The latter is sufficient to support transcription of 10,000 somatic nuclei in the early embryo (Bellier et al., 1997), and we sought to determine the dynamics of B4 binding and Pol II phosphorylation during nuclear reprogramming. We carried out time course analyses at the single-nucleus level following MEF NT (Figure 2A). Transplanted nuclei were fixed 0–1, 6, 24, and 48 hr after NT and immunostained. The specificity of the antibodies used has been extensively tested and shown to work in the *Xenopus* oocyte GV (Figure S2A) (Doyle et al., 2002, Gall and Murphy, 1998, Jullien et al., 2010, Morgan et al., 2000, Palancade et al., 2001, Roth et al., 1990, Xie et al., 2006). Consistent with a previous study, oocyte-specific B4 was rapidly recruited to transplanted nuclei, with over 80% of the transplanted nuclei strongly bound by B4 within 24 hr (Figures 2B and 2C; Table S3) (Jullien et al., 2010). The Pol II catalytic subunit RPB1 in its hypophosphorylated form (Pol IIA) was also rapidly recruited to transplanted nuclei, with kinetics closely following those of B4 (Figures 2B and 2C; Table S3). Phosphorylation of RPB1 was induced specifically within a subset of the Pol IIA marked nuclei. The proportion of nuclei showing RPB1 phosphorylated on serine 5 (Ser5P Pol II, initiation type) and on serine 2 (Ser2P Pol II, elongation type) increased gradually and sequentially over time (Figures 2B–2D; Table S3). Real-time confocal imaging of transplanted nuclei using fluorescently labeled antigen binding fragments against specific phosphorylated RPB1 isoforms confirmed that most transplanted nuclei showed phosphorylated RPB1 (Hayashi-Takanaka et al., 2011) (Figures S2B–S2F). These results indicate that transcriptional reprogramming by *Xenopus* oocytes is uniform over time and characterized by the efficient recruitment and phosphorylation of Pol II to the great majority of transplanted nuclei within 48 hr.

To further define the sequence of events leading to transcriptional reprogramming by the oocyte, we determined temporal correlations between pairs of markers at the single-nucleus level. In time-course experiments, B4 staining was detected in a few nuclei that were not costained by RPB1 antibodies (Figure 2E, arrows). In contrast, nuclei labeled by hypophosphorylated Pol IIA were always positive for B4 (Figure 2F, arrowheads), indicating that Pol IIA recruitment follows B4. The proportion of B4 nuclei not marked by Pol IIA was low at 6 hr onward (<5%, 42 of 44 B4+ nuclei were also Pol IIA+; Figures 2F and 2G; Table S3), suggesting that Pol IIA closely follows B4 binding during nuclear reprogramming. Pol IIA binding in transplanted nuclei was first seen in the absence of RPB1 phosphorylation (Figure 2F, arrowheads), but all nuclei marked by Ser5P or Ser2P Pol II also had Pol IIA and B4 (Figure 2F, big arrows, and Figure 2G). When Ser2P Pol II was present, all other markers were also seen in transplanted nuclei (Figure 2G; data not shown). Taken together, our time-course analysis at the single-nucleus level reveals a sequence of events compatible with a hierarchical model in which B4 binding precedes Pol IIA recruitment, which is then followed by phosphorylation of the C-terminal domain (CTD) of RPB1 on serine 5 then serine 2 in most of transplanted nuclei over time.

### Somatic Transcriptional Components Are Lost and Oocyte Counterparts Are Gained during Reprogramming

**Figure 3:**
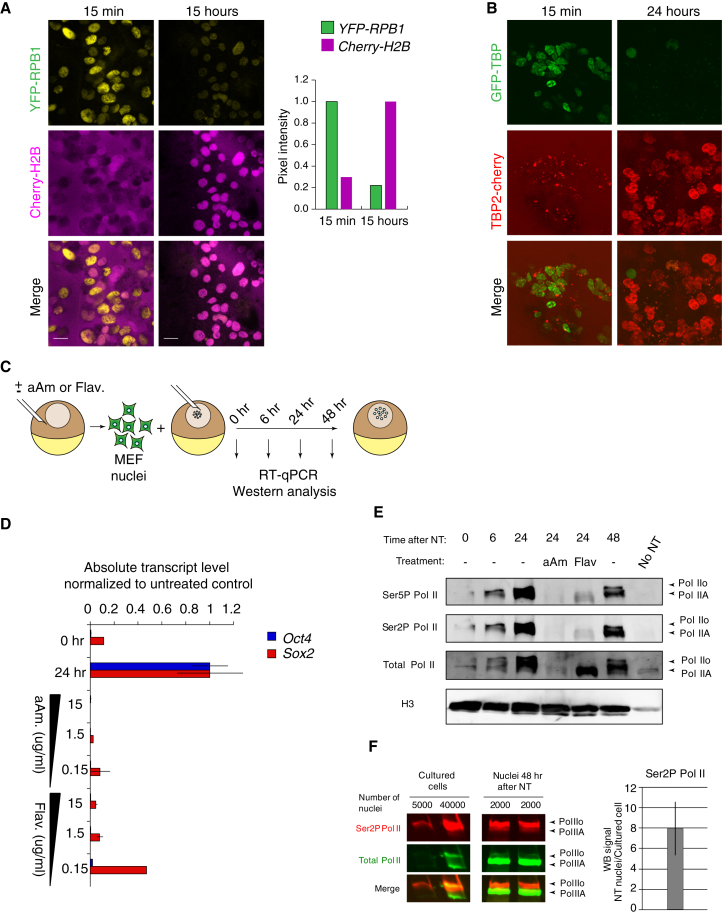
An Exchange from Somatic to Oocyte Transcriptional Machinery Takes Place during Reprogramming by the *Xenopus* Oocyte (A) Confocal imaging of YFP-RPB1 expressing nuclei (yellow) transplanted into oocytes expressing cherry-H2B (magenta). Images were recorded 15 min and 15 hr after NT. Scale bar, 20 μm. Graph at right: average mean pixel intensity per nucleus normalized to the highest value. (B) GFP-TBP nuclei (green) transplanted into oocytes expressing TBP2-cherry (red). Confocal images were recorded soon after (15 min) and 24 hr after NT. Incorporation of TBP2-cherry and disappearance of GFP-TBP are observed. (C) Experimental set-up for western blot analysis and transcriptional inhibition with aAm or Flav. (D) Transcriptional inhibition by aAm and Flav inhibits activation of *Oct4* and *Sox2* in NT oocytes, as examined by RT-qPCR analyses. n = 3. Data are represented as mean ± SEM. (E) Western blot analysis of total Pol II, which recognizes both Pol IIA (hypophosphorylated) and Pol IIo (hyperphosphorylated), Ser5P Pol II, Ser2P Pol II, and histone H3 in MEF nuclei 0, 6, 24, and 48 hr after NT. Arrowheads indicate Pol IIA and Pol IIo. aAm and Flav treatments of transplanted nuclei are shown. (F) Comparison of the Ser2P Pol II band intensity (red) before and after NT. The band intensity, detected by western blot, was normalized to the number of nuclei. Fold enrichment of Ser2P Pol II in NT samples over donor cells is shown in the graph. Data are represented as mean ± SEM; n = 3. See also Figure S3 and Movies S1 and S2.

We next tested if this extensive, selective, and hierarchical reprogramming of somatic nuclei by the *Xenopus* oocyte may reflect an exchange of somatic-to-oocyte transcriptional machinery. Using yellow fluorescent protein (YFP)-RPB1 donor nuclei (Darzacq et al., 2007) to determine the fate of somatic Pol II during reprogramming, we found that somatic YFP-RPB1 is lost from transplanted nuclei within 15 hr after NT (Figures 3A and S3A; Movie S1). Somatic RPB1 disappearance from transplanted nuclei coincided with the incorporation of histone cherry-H2B from the oocyte, which marks transplanted nuclei (Figure 3A). Given the high RPB1 increase in transplanted nuclei (Figures 2 and S2), our results are consistent with an exchange from somatic to oocyte RPB1 in transplanted nuclei during reprogramming.

We next tested whether the general somatic transcription factor TATA-binding protein (TBP), important for the formation of a preinitiation complex, is exchanged for oocyte TBP2 during reprogramming (Akhtar and Veenstra, 2009). GFP-TBP-expressing nuclei (de Graaf et al., 2010) were transplanted into *Xenopus* oocytes preloaded with TBP2-cherry (Jallow et al., 2004). Donor GFP-TBP in somatic nuclei was replaced by oocyte TBP2-cherry within 24 hr after NT (Figures 3B and S3B; Movie S2). We conclude that both somatic RPB1 and somatic TBP are lost from transplanted nuclei and replaced by their oocyte counterparts after NT.

### Nuclei Undergoing Reprogramming Show Highly Abundant Loading of Phosphorylated RPB1

Transcription in *Xenopus* oocytes is characterized by an extremely high rate of transcription (Callan, 1982, Davidson, 1986). We therefore asked if transplanted nuclei, which were loaded with the oocyte transcription machinery, contained unusually high levels of RPB1 phosphorylation. To gain a quantitative view of RPB1 phosphorylation during reprogramming, we transplanted MEF nuclei to oocytes and reisolated transplanted nuclei at several time points after NT followed by western blot analysis to probe RPB1 chromatin recruitment (Figure 3C) (Murata et al., 2010). We used, as controls, transcriptional inhibitors alpha-Amanitin (aAm), which binds with high specificity and high affinity near the catalytic site of RPB1, preventing transcript synthesis, and leading to RPB1 degradation in cultured cells (Bensaude, 2011); and Flavopiridol (Flav), which prevents productive elongation of transcription by inhibiting, among several kinases, the kinase activity of CDK9, the catalytic subunit of pause-release factor P-TEFb (Figure 3D) (Chao and Price, 2001). Consistent with our immunostaining time course (Figure 2), hypophosphorylated RPB1 was strongly recruited to transplanted chromatin, migrating as a 214 kDa band (Palancade et al., 2001) (Figure 3E, Pol IIA). Remarkably, RPB1 phosphorylation was strongly induced in as little as 24 hr, giving a band of around 240 kDa (Palancade et al., 2001) (Figure 3E, Pol IIo). aAm treatment fully abolished Ser2P Pol II, Ser5P Pol II, and Total total Pol II (Figure 3E). Flav also inhibited RPB1 phosphorylation. We sought to precisely estimate the amount of active Pol II in transplanted nuclei as compared to cultured cells. For this purpose, we analyzed by western blot cell samples directly lysed from cultured dishes and from transplanted nuclei extracted immediately after isolation of GVs in order to minimize changes in phosphorylation due to technical manipulation. It is striking that the level of phosphorylated RPB1 (Ser2P Pol II) was, on average, 8-fold higher in transplanted nuclei compared to somatic nuclei before NT (Figure 3F). We conclude that NT to the *Xenopus* oocyte entails efficient recruitment of RNA Pol II to transplanted nuclei to reach an unusually high level of phosphorylation on RPB1 CTD.

### Oocyte Pol II Is Required for Reprogramming

**Figure 4:**
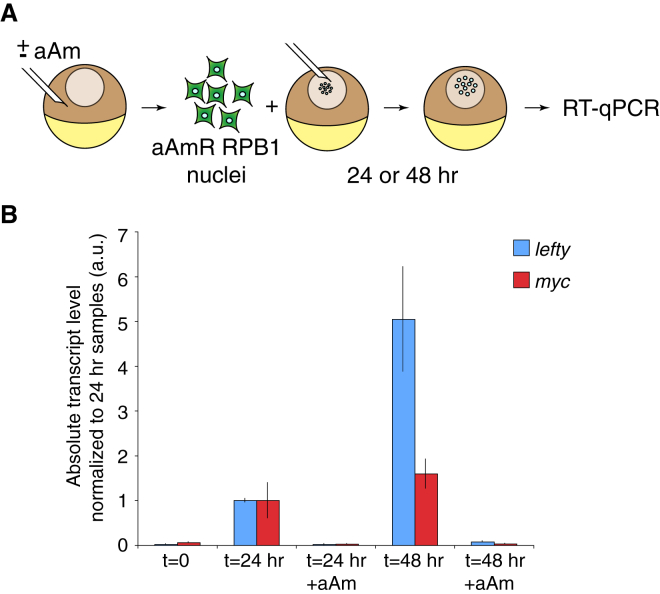
Somatic RPB1 Is Not Sufficient for Reprogramming by the Oocyte (A) NT of aAmR RBP1 somatic cell nuclei. aAmR-RPB1 murine erythroleukemia nuclei were transplanted into oocytes in the absence or in the presence of transcriptional inhibitor aAm. (B) Quantitative analysis of *Lefty* (blue) and *Myc* (red) transcripts in oocytes transplanted with aAmR-RPB1 nuclei and cultured in the presence or in the absence of aAm for 0, 24, or 48 hr. Error bars indicate SEM. a.u., arbitrary unit. See also Figure S4.

The exchange in transcriptional machinery in transplanted nuclei suggests that somatic RPB1 may not be sufficient for the reprogramming of transplanted nuclei and that a contribution from oocyte Pol II may be required. To test this rigorously, we transplanted mouse erythroleukemia nuclei expressing an alpha-Amanitin resistant (aAmR) form of RPB1 (Custódio et al., 2006). We confirmed these cells to be aAmR (Figure S4). The recipient oocytes were preinjected with aAm (Figure 4A). Whereas the transcriptional reactivation of mouse *Lefty1* and *c-myc* was detected in these transplanted erythroleukemia nuclei 24 hr and 48 hr after NT, aAm (which inhibits *Xenopus* RPB1 but not somatic RPB1) prevented gene reactivation, even in the presence of aAmR donor somatic RPB1 (Figure 4B). Thus, somatic RPB1 is not sufficient for nuclear reprogramming by the *Xenopus* oocyte, which must, therefore, depend on RPB1 derived from the oocyte.

### Oocyte Linker Histone Binding to Transplanted Chromatin

**Figure 5:**
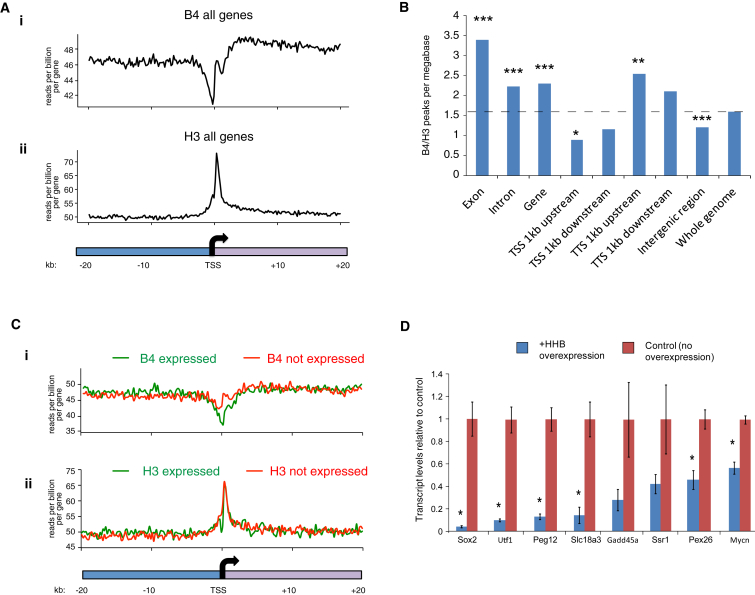
Genome-wide Oocyte Linker Histone B4 Binding to the Chromatin of Transplanted Nuclei (A) The binding of B4 was analyzed in the ±20 kB region surrounding the TSS of all mouse genes. The graph shows the normalized read count for B4 (top [i]) and H3 (bottom [ii]) across this region. The longitudinal axis shows reads per billion per gene in each bin. (B) Distribution of B4 peaks in different genomic regions. B4/H3 peaks per megabase were calculated across different genomic regions. The dotted line shows the whole genome average value. Statistical significance was evaluated against peaks in the whole genome. p values were calculated using t test. ^∗∗∗^p < 0.0001; ^∗∗^p < 0.001; and ^∗^p < 0.01. (C) Same as in (A) but for the genes that are expressed (green) and not expressed (red) after NT (as determined by RNA-seq analysis in Figure 1B). (D) HHB overexpression impairs activation of reprogrammed genes that were identified by RNA-seq analysis. Gene reactivation in NT oocytes was judged by RT-qPCR analysis. Data are represented as mean ± SEM; n = 4. p values were calculated using ANOVA; ^∗^p < 0.01. See also Figure S5.

Since oocyte-specific linker histone B4 is the earliest identified oocyte factor to bind to transplanted nuclei in the reprogramming sequence (Figure 2B), we investigated its precise binding pattern on somatic chromatin in relation to transcriptional reprogramming. We thus generated genome-wide high-resolution binding maps of B4 in transplanted nuclei. We successfully carried out ChIP-seq analyses for histone B4 and core histone H3 from oocytes 48 hr after NT of MEF nuclei. One advantage of this procedure is that it allows a distinction between ChIP-seq reads from control *Xenopus* follicle cells, hundreds of which surround each oocyte, and those of mouse chromatin coming from transplanted nuclei. We therefore compared sequence reads that mapped either to the mouse or *Xenopus* genome. We found that 73% of the H3 ChIP-seq reads mapped to the mouse genome, while 27% to the *Xenopus* genome. In contrast, 97% of B4 ChIP-seq reads mapped to the mouse genome, consistent with the exclusive expression of B4 in the oocyte and not in follicle cells, a result that confirms the specificity of the B4 antibody in ChIP analysis. In general, the binding of B4 to transplanted mouse chromatin was widespread across the mouse genome. However, B4 was depleted at transcription start sites (TSSs) compared to adjacent regions (Figure 5Ai). This was not due to the lack of nucleosomes at TSSs, since, unlike B4, H3 was enriched where B4 was depleted around TSSs (Figures 5Aii and S5A). We defined 6,141 peaks of significant enrichment of B4 over H3 (hereinafter called B4/H3 peaks). These were significantly underrepresented around TSSs in contrast to transcription termination sites (TTSs) (Figure 5B), in good agreement with metaplots data (Figure 5A). It is interesting that B4/H3 peaks were significantly enriched in exons over the whole genome average (Figures 5B and S5B). Enrichment of B4/H3 peaks in exons differs from the somatic type linker histone H1 distribution, while depletion around TSSs is strikingly similar (Cao et al., 2013). Somatic type H1 is involved in gene repression, while B4 is important for transcriptional reprogramming (Jullien et al., 2010). Therefore, the differential binding properties of H1 and B4 might reflect functional differences between these two linker histones.

To determine the relationship between B4 depletion at the TSS and gene expression after NT, we compared B4 binding at the TSS of expressed versus nonexpressed genes after NT as detected by RNA-seq (Figure 1B). Depletion of B4 at the TSS was more pronounced for active genes than for inactive genes (Figure 5Ci; TSS ± 2 kb, p < 5.9 × 10^−5^), in good agreement with the linker histone H1 binding pattern described in mouse ESCs (Cao et al., 2013). In conclusion, oocyte linker histone B4 is distributed throughout chromatin of transplanted nuclei during reprogramming and B4 depletion around TSSs is a hallmark of actively transcribed genes. Thus, B4 binding reflects the transcriptional state of individual genes after NT.

Finally, we asked whether B4 binding to chromatin is required for transcriptional reprogramming. We used a dominant negative form of B4, HHB, which has been shown to inhibit B4 binding to chromatin (Jullien et al., 2010). We examined the effect of HHB overexpression on transcription of several genes that were identified by RNA-seq analyses as expressed after NT. HHB overexpression in NT oocytes significantly inhibited transcription from most of reprogrammed genes tested (6/8) (Figure 5D, p < 0.01). In summary, the early reprogramming event described here, namely oocyte-specific histone B4 loading to somatic chromatin, is required for successful transcriptional reprogramming.

## Discussion

Here, we have used a combination of genome-wide and single-nucleus-level analyses to provide a comprehensive view of somatic cell reprogramming following NT to the *Xenopus* oocyte. Our results define a hierarchical sequence of events leading to rapid, specific, and genome-wide reprogramming of transcription and provide molecular and mechanistic insights into this process, as well as a valuable resource for future studies.

One important outcome of our analysis is that it overturns the view that transcriptional reprogramming by NT to *Xenopus* oocytes leads to unspecific transcription of all genes. This view originated from the observation that, in addition to pluripotency genes, differentiated cell-type-specific genes can be reactivated following oocyte NT (Biddle et al., 2009), as well as from the fact that transcription of lampbrush chromosomes is widespread and characterized by an extremely high rate of transcription, with transcription of differentiation-related genes (Callan, 1986, Davidson, 1986, Gall, 1954, Simeoni et al., 2012). However, our RNA-seq analysis demonstrates that many genes are rapidly and reproducibly downregulated and upregulated after NT of mouse fibroblast nuclei (Figure 1B), indicating that reprogramming by the oocyte is selective. Moreover, the oocyte induces a preferential transcription of mouse orthologs of *Xenopus* genes highly expressed in oocytes, distinct from a pluripotency pattern. Thus, the oocyte system induces a genome-wide, selective shift in transcription toward an oocyte pattern rather than a pluripotent stem cell one. This makes an important distinction between the *Xenopus* oocyte NT system and other reprogramming methods such as reprogramming to iPSCs. While splicing events and polyadenylation are clear evidence for RNA Pol II-mediated transcription, we recognize that our analysis is focused on polyadenylated transcripts. We cannot exclude the possibility that the extent of reprogramming may even be far greater than seen here, as many nonpolyadenylated transcripts may also be produced after NT. It will be interesting to define and compare the sequence of molecular events that take place after NT in the mouse. Future studies are also required to determine the relationship between the epigenetic state of donor nuclei and the selective transcription seen after NT. The *Xenopus* oocyte NT system combined with RNA-seq analysis of transcripts described here provides a convenient model to study how the epigenome of different donor cell types influences reprogramming.

**Figure 6:**
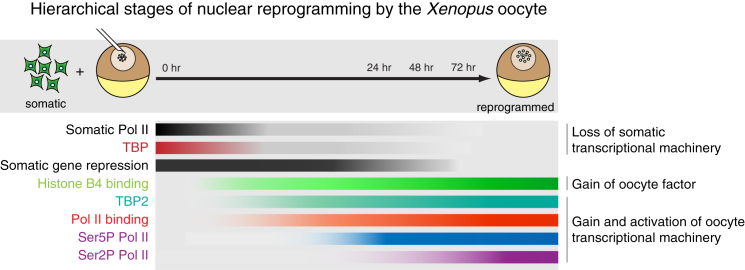
Hierarchical Sequence of Events of Transcriptional Reprogramming by the *Xenopus* Oocyte Stages of nuclear reprogramming by the *Xenopus* oocyte. After NT to the *Xenopus* oocyte, somatic nuclei are bound by oocyte-specific B4. Loss of somatic Pol II and TBP starts. B4 binding is followed by the accumulation of oocyte-specific TBP2 and hypophosphorylated RPB1 (Pol II binding) derived from the oocyte, which becomes subsequently phosphorylated on serine 5. This is followed by serine 2 phosphorylation, resulting in productive transcription. The same ordered sequence of events takes place in most of transplanted nuclei within 48 hr, with slightly different kinetics.

To our knowledge, *Xenopus* oocyte NT is probably a unique reprogramming system in which genome-wide transcription can rapidly and extensively be induced in the absence of protein synthesis, indicating that all of the reprogramming factors are present in the oocyte at the time of NT. In our time course, single-nucleus analyses reveal a hierarchical binding and activation of oocyte-derived components accompanied by the replacement of somatic factor counterparts. Binding of oocyte linker histone B4 to transplanted nuclei precedes oocyte Pol II loading, ultimately leading to intense Pol II activation on transplanted somatic chromatin (Figure 6). This probably results from the overwhelming abundance of oocyte factors compared with somatic ones. Components such as oocyte TBP2 and oocyte RPB1 replace their somatic equivalents in transplanted nuclei, suggesting that oocyte reprogramming entails a shift in basal transcriptional machinery. Moreover, the hierarchical sequence of molecular events takes place in the great majority of transplanted nuclei within 2 days, a process that represents an unusually high reprogramming efficiency. This high efficiency is the likely result of the rapid exchange of basal transcriptional machinery. Interestingly, recent work has challenged the view that the basal transcriptional machinery is universal and highly conserved in eukaryotic cells, in agreement with the idea that change in its key components can mediate cell-type-specific transcription (Akhtar and Veenstra, 2011, Goodrich and Tjian, 2010). Given that basal transcription factors TBP2 and TAF4b play key roles in transcription of oocyte-specific genes (Akhtar and Veenstra, 2009, Freiman et al., 2001, Gazdag et al., 2009, Geles et al., 2006), we propose that an exchange in basal transcription machinery mediates reprogramming by the *Xenopus* oocyte. Other oocyte factors, such as histone B4 (Saeki et al., 2005), nucleoplasmin (Tamada et al., 2006), nuclear actin and Wave1 (Miyamoto et al., 2011, Miyamoto et al., 2013), and histone H3.3 (Jullien et al., 2012), may modify somatic chromatin to make it accessible to the oocyte transcriptional machinery, thus enabling unusually high amounts of Pol II loading.

Several species, including *Xenopus*, mouse, and *Drosophila*, possess oocyte-specific variants of linker histone H1 (Pérez-Montero et al., 2013, Smith et al., 1988, Tanaka et al., 2001). Their involvement in nuclear reprogramming has been reported (Gao et al., 2004, Jullien et al., 2010, Teranishi et al., 2004). The ectopic expression of H1foo, the mouse homolog of *Xenopus* B4, prevents the exit from the pluripotent state in mouse ESCs (Hayakawa et al., 2012) although the overexpression of H1foo did not improve iPSC formation (Shinagawa et al., 2014). In mouse embryos, H1foo quickly replaces somatic H1 immediately after NT (Teranishi et al., 2004). However, how oocyte-linker histone contributes to reprogramming of somatic nuclei has remained unclear. Here, our genome-wide analysis of B4 binding revealed pervasive binding of B4 to chromatin of transplanted nuclei. We envisage a progressive transition during reprogramming in oocytes (Figure 6). First, a widespread binding of oocyte linker histone B4 to chromatin happens, while somatic linker histones are removed. As a next step, B4 around TSS is evicted due to the binding of H3.3 (Braunschweig et al., 2009). The specific binding property of B4, compared to H1, allows eviction by H3.3. Finally, transcription is driven by abundant oocyte Pol II. Oocyte linker histone B4 is less positively charged than its somatic counterpart H1, hence exhibiting weaker interaction with DNA. In fact, *Xenopus* linker histone B4 has been shown to be more mobile than H1 (Jullien et al., 2010) and promotes chromatin accessibility to remodeling factors (Saeki et al., 2005). Therefore, genome-wide binding of histone B4 should make somatic chromatin accessible to other oocyte factors. This widespread loosening of somatic chromatin may help oocyte transcription factors to gain access to gene regulatory regions, promoting formation of a preinititation complex containing oocyte Pol II. It is also possible that there are oocyte-specific transcription factors ultimately driving the expression of the oocyte program, so that there may be more specificity to the *Xenopus* oocyte transcriptional reprogramming system than can currently be explained. Such transcriptional activators may support efficient reprogramming as iPSC-mediated reprogramming can be boosted by enhancing transcriptional activation (Di Stefano et al., 2014, Pijnappel et al., 2013). Nevertheless, once transcription initiation complexes are assembled at promoters, productive transcription then may lead to, and is supported by, incorporation of histone H3.3 (Skene and Henikoff, 2013). Indeed, H3.3 incorporation into somatic chromatin has been shown to represent a critical step toward transcriptional reprogramming in *Xenopus* oocytes (Jullien et al., 2012). It is interesting that H3.3 also inhibits linker histone binding to chromatin in somatic cells (Braunschweig et al., 2009). Therefore, displacement of B4 around the TSSs of active genes 48 hr after NT can be explained by abundant H3.3 incorporation at these regions. These stepwise molecular processes at TSSs in reprogrammed genes (B4 incorporation; H3.3 incorporation; and, finally, Pol II activation) can lead to rapid, direct reprogramming.

In summary, the induction of an oocyte program of transcription seems to prepare a transplanted somatic nucleus for a major switch to many different gene expression options during cell differentiation. Overall, our study supports the deterministic reprogramming model in oocytes (Jullien et al., 2011) and provides a fruitful basis to dissect the mechanisms of the battle between the oocyte factors that induce reprogramming and those of somatic chromatin that resist reprogramming by oocyte factors.

## Experimental Procedures

### NT

Donor cells were permeabilized with streptolysin O, and approximately 300 permeabilized cells were injected into the GV of *Xenopus* oocytes (Halley-Stott et al., 2010). NT oocytes were incubated at 18°C. Inhibition of translation was carried out by adding 10 μg/ml CHX to the oocyte culture medium. Culture with CHX was started a few hours before NT and continued for 2 days until samples were collected. For transcriptional inhibition experiments, 15 nl of 1,000 μg/ml, 100 μg/ml, 10 μg/ml aAm or Flav solution, or H_2_O control, was injected into the GV to give final aAm concentrations of 15 μg/ml, 1.5 μg/ml, or 0.15 μg/ml, respectively. aAm and Flav, with a final concentration of 1.5 μg/ml, were used for subsequent inhibition experiments. mRNA injections were as follows: 2.3 ng Cherry-H2B, 13.8 ng TBP2-cherry, 13.8 ng HHB (Jullien et al., 2010). All experimentation with frogs was carried out following requirements of the UK Home Office.

### Immunofluorescence and Live Cell Imaging

GVs containing transplanted nuclei were dissected and fixed immediately in 4% paraformaldehyde/1× PBS overnight at 4°C, stained using primary and secondary antibodies (as detailed in the Supplemental Experimental Procedures), and imaged using confocal microscopy. For live imaging, GVs containing transplanted nuclei were isolated under mineral oil and imaged using confocal microscopy.

### Quantitative RT-PCR

Quantitative RT-PCR (RT-qPCR) analyses were performed to detect reprogrammed transcripts from transplanted mouse nuclei. Four whole injected oocytes were pooled as one sample, and RNA was extracted from the samples using QIAGEN RNeasy columns. After RNA extraction including on-column DNase I digestion, reverse transcription was performed using SuperScript III with gene specific primers. Real-time PCR was performed as SYBR Green assays on an ABI 7300 Real Time PCR Cycler using a standard ABI cycling condition. Primers used in this assay are shown in the Supplemental Information.

### RNA Immunoprecipitation

BrUTP (Sigma: B7166, 4.6 nl of 100 mM stock) was injected to the cytoplasm of *Xenopus* oocytes 2 hr after transplantation with MEFs. Oocytes were collected 48 hr after NT, and RNA was extracted using a QIAGEN RNeasy kit (eight oocytes per column). RNA immunoprecipitation (RIP) was performed using a protocol adapted from the previous report (Core and Lis, 2008): BrUTP-labeled RNAs from 16 NT oocytes were immunoprecipitated by mixing with 20 μl of anti-bromodeoxyuridine (anti-BrdU) agarose conjugate (Santa Cruz Biotechnology: sc-32323-AC, blocked overnight in 0.5× saline-sodium phosphate-EDTA [SSPE] buffer supplemented with 0.05% Tween 20, 0.1% polyvinylpyrrolidone [PVP], and 1 mg/ml BSA) and 500 μl RIP buffer (0.5× SSPE with 0.05% Tween 20 and RNase inhibitor) and incubating for 4 hr at 4°C on a rotating wheel. Agarose conjugates were then washed once with low salt buffer (0.2× SSPE with 0.05% Tween 20), twice with high salt buffer (0.5× SSPE with 0.05% Tween 20 and 150 mM NaCl), and once with TET buffer (10 mM Tris, 1 mM EDTA, pH 8, and 0.05% Tween 20). Immunoprecipitated RNAs were then eluted from the agarose beads by incubating for 5 min at room temperature in 133 μl elution buffer (300 mM NaCl, 5 mM Tris, pH 7.5, 1 mM EDTA, 0.1% SDS, 20 mM dithiothreitol). The elution is repeated two more times. A total 400 μl of eluted RNAs were then extracted with phenol/chloroform, ethanol precipitated, and then resuspended in water before proceeding to RNA-seq libraries production.

### RNA-Seq

Newly transcribed RNAs in NT oocytes were isolated by RIP. These RNAs were reverse-transcribed and complementary DNAs (cDNAs) amplified following a published protocol (Tang et al., 2010). Amplified cDNAs with a size between 0.5 and 3 kb were collected, and 50 ng of DNA was sonicated in a Bioruptor TWIN (7 min twice with a 30 s/30 s on/off cycle, medium strength). Libraries were made from the sonicated DNA using reagents provided in the Illumina TruSeq DNA Sample Prep Kit (FC-121-2001). Ten nanograms of DNA was subjected to end repair and then purified using the MinElute PCR Purification Kit (QIAGEN). After A tailing, appropriate adapters were ligated. DNA with adapters was purified using Agencourt AMPure XP beads (Beckman Coulter). DNA was amplified by 16–20 PCR cycles. Size selection of PCR products was carried out using AMPure beads (selection of fragments between 250 and 370 base pairs). Libraries were validated by Tape station (Agilent) and were sequenced on Illumina HiSeq 2000.

### ChIP Analysis

Details of ChIP analysis of NT oocytes were described elsewhere (Miyamoto et al., 2011). Briefly, a set of seven NT oocytes, which are equivalent to ∼2,100 mouse nuclei, was transferred into a 1.5 ml tube. NT oocytes were crosslinked for 10 min at room temperature in 1 ml of MBS medium containing 1% formaldehyde. After three quick washes, the oocytes were ruptured in 280 μl of homogenization buffer. Sonication was carried out in 1.5 ml tubes for 7 min twice with a 30 s/30 s on/off cycle on Bioruptor TWIN (Diagenode). Sonicated samples were diluted with buffer to adjust SDS concentration (0.1% in final concentration). After centrifugation, supernatants were transferred as chromatin solutions. The chromatin solution was mixed with an antibody and incubated overnight at 4°C with rotation. After the antibody incubation, 20 μl of dynabeads protein G (Invitrogen) was added and rotated for another 6 hr at 4°C, followed by several washes. Finally, crosslinking was reversed and DNAs were isolated.

### ChIP-Seq

For ChIP-seq analyses, 364 and 140 NT oocytes were used for histone B4 and H3 library preparation, respectively. Antibodies used include a rabbit polyclonal B4 antibody (Ohsumi et al., 1993) and a rabbit polyclonal anti-histone H3 antibody (ab1791, Abcam). DNA fragments obtained from ChIP pulldowns were subjected to *E. coli* DNA polymerase I (New England Biolabs, M0209) treatment before end repair reaction. The samples were then amplified by following the library preparation protocol described for RNA-seq (20 PCR cycles). The ChIP-seq libraries were sequenced on an Illumina HiSeq 2000.

### Western Blot

Western blots were performed following standard protocols. Anti-mouse, -rabbit, or -goat immunoglobulin G Alexa Fluor 680 (Invitrogen) and/or anti-mouse or -rabbit IRDye 800CW (LICOR) were used as a secondary antibody, and bands were detected using the LI-COR ODYSSEY imaging system. Primary antibodies used were as follows: rabbit polyclonal anti-Pol II CTD repeat YSPTSPS (phosphoS2) (ab5095, Abcam), mouse monoclonal anti-Pol II CTD repeat YSPTSPS (8WG16) (ab817, Abcam), mouse monoclonal anti-HA (H9658, Sigma).

### Bioinformatics

See Supplemental Information.

### Cell Culture

MEFs were derived from embryos (embryonic day 13.5) hemizygous for the X-GFP transgenic allele (Hadjantonakis et al., 2001) as described elsewhere (Pasque et al., 2011). MEFs were immortalized following SV40 Large T Antigen expression. The YFP-RPB1 U2OS line was characterized in (Darzacq et al., 2007). The aAm-resistant murine erythroleukemia cell line (clone 8) was described in Custódio et al. (2006).

### Statistical Analysis

The number of biological replicates are shown as n. In transcriptional assays by RT-qPCR, the statistical difference was calculated by ANOVA. Error bars represent SEs. Statistical tests in RNA-seq and ChIP-seq analyses are described in Supplemental Information.

## Author Contributions

J.J., K.M., V.P., and J.B.G. designed experiments, analyzed data, and performed almost all experiments. V.P., K.M., and J.B.G. wrote the manuscript with help from J.J. G.E.A. and C.R.B. analyzed genome-wide data. R.P.H. performed experiments. N.J.G., H.K., and K.O. generated reagents.

## Accession Numbers

The accession number for the RNA-seq and ChIP-seq data reported in this article is SRP042359 (National Center for Biotechnology Information Sequence Read Archive database).

## Supplemental Information

Document S1. Supplemental Experimental Procedures and Figures S1–S5

Table S1. RNA-Seq Analysis of Mouse Nuclei Before and After NTRelated to Figure 1

Table S2. RNA-Seq Analysis of Untransplanted Xenopus OocytesRelated to Figure 1

Table S3. Proportion of MEF Nuclei Showing Histone B4 Loading, Pol II Recruitment, Ser5P Pol II (Initiation), and Ser2P Pol II (Elongation) after NT to Xenopus OocytesRelated to Figure 2. The number and proportion of nuclei stained by antibodies against histone B4, Pol IIA, Ser5P Pol II, and Ser2P Pol II is indicated. An asterisk denotes the number of DAPI nuclei counted in the Ser2P Pol II immunostaining

Movie S1. Somatic Pol II Is Lost from Transplanted Nuclei Related to Figure 3. Somatic RPB1 is lost after NT. Confocal time-lapse imaging of YFP-RPB1 U2OS nuclei transplanted into oocytes expressing Cherry-H2B. Confocal images were recorded every 20 min for 12 hr, starting 15 min after NT. YFP-RPB1 is shown in green, and Cherry-H2B is shown in red. Images are projected Z sections. Scale bar, 20 μm. Still images at various time points are shown in Figure S3A.

Movie S2. Somatic TPB Is Lost from Transplanted Nuclei Related to Figure 3. Confocal time-lapse imaging of GFP-TBP U2OS nuclei transplanted into oocytes. Confocal images were recorded every 3,700 s overnight, starting 15 min after nuclear transfer. Images are projected Z sections. Still images are shown in Figure S3B.

Document S2. Article plus Supplemental Information
