# Supplementary material for: Hierarchical Molecular Events Driven by Oocyte-Specific Factors Lead to Rapid and Extensive Reprogramming
Source: Mol Cell. 2014 Aug 21;55(4):524–36. doi: 10.1016/j.molcel.2014.06.024 (PMC4156308; doi:10.1016/j.molcel.2014.06.024)
Supplement: Document S1. Supplemental Experimental Procedures and Figures S1–S5 [file mmc1.pdf]

Molecular Cell, Volume *55*

Supplemental Information

Hierarchical Molecular Events Driven by Oocyte-Specific Factors Lead to Rapid and Extensive Reprogramming

Jerome Jullien, Kei Miyamoto, Vincent Pasque, George E. Allen, Charles R. Bradshaw, Nigel J. Garrett, Richard P. Halley-Stott, Hiroshi Kimura, Keita Ohsumi, and John B. Gurdon

SUPPLEMENTAL FIGURES AND LEGENDS

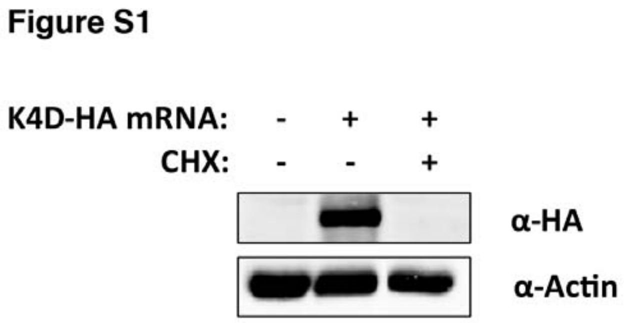

Figure S1. Protein translation is inhibited by cycloheximide (CHX) treatment in *Xenopus* oocytes (related to figure 1).

Kdm4d mRNA tagged with HA was injected into *Xenopus* oocytes and the injected oocytes were cultured in the medium with or without the presence of CHX. Translation of injected mRNA was examined by Western blot, using an antibody against HA. Actin was used as a loading control.

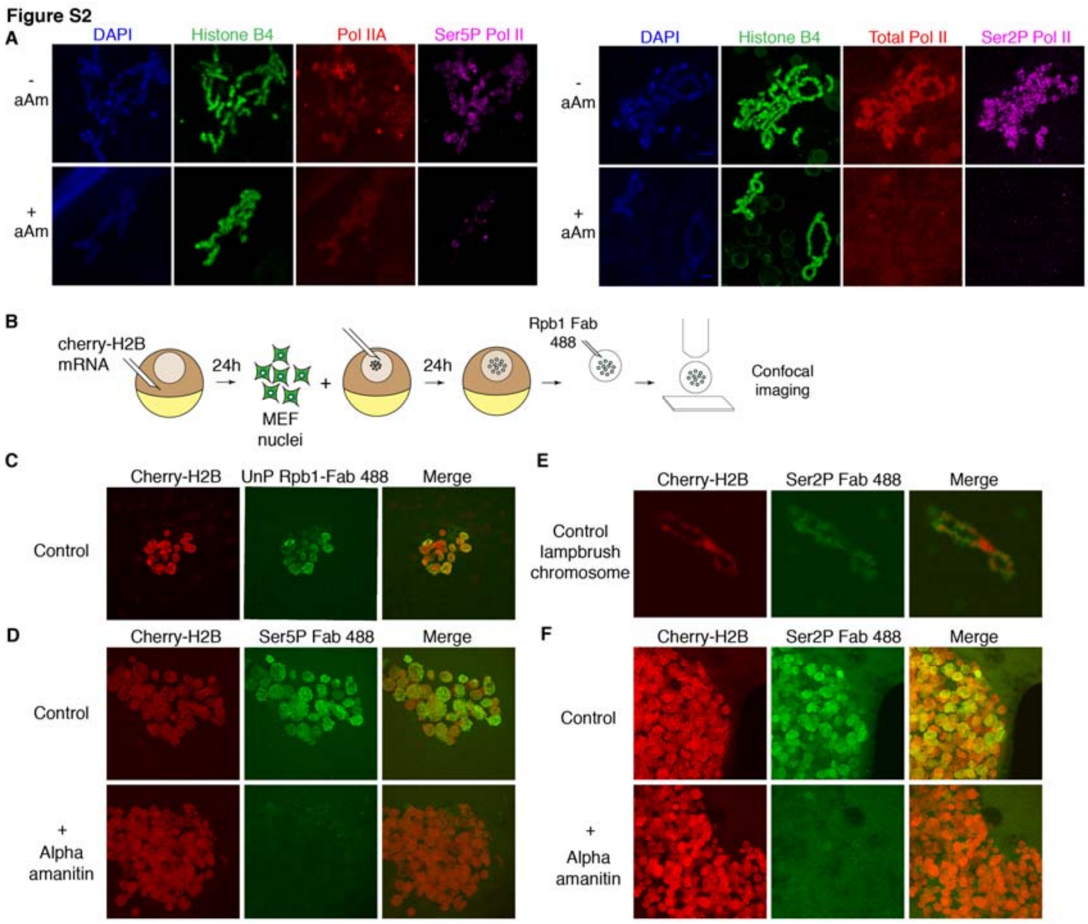

Figure S2. Phosphorylated RNA polymerase II is recruited to most of transplanted nuclei (related to figure 2).

(A) Antibody specificity control on *Xenopus* lampbrush chromosomes. Confocal images of lampbrush chromosomes immunostained for B4 (green), Pol IIA (red in left), Ser5P Pol II (magenta in left), total Pol II (red in right), Ser2P Pol II (magenta in right) in the absence or in the presence of transcriptional inhibitor alpha-Amanitin (aAm) for 24 hr. Transcriptional inhibition by alpha-Amanitin (aAm) efficiently inhibits Pol II recruitment and phosphorylation of lampbrush chromosomes. Total Pol II includes hypo- and hyperphosphorylated Pol II. DAPI is in blue. Images are projected Z-sections. Scale bar = 5 μm.

(B) Experimental scheme for confocal live cell imaging of injected fluorescently labeled specific antigen binding fragment (Fab) against un- or phosphorylated RPB1. Cherry-H2B mRNA injection to oocytes was used to label transplanted mouse nuclei.

(C) Confocal image of transplanted MEF nuclei using Fab Alexa488 against unphosphorylated RPB1.

(D) Confocal image of transplanted MEF nuclei using Fab Alexa488 against Ser5P RPB1. Alpha-Amanitin (aAm) inhibits Ser5P RPB1 recruitment.

(E) Confocal image of a lampbrush chromosome using Fab Alexa488 against Ser2P RPB1

(F) Confocal image of transplanted MEF nuclei using Fab Alexa488 against Ser2P RPB1. Alpha-Amanitin (aAm) inhibits Ser2P RPB1 recruitment.

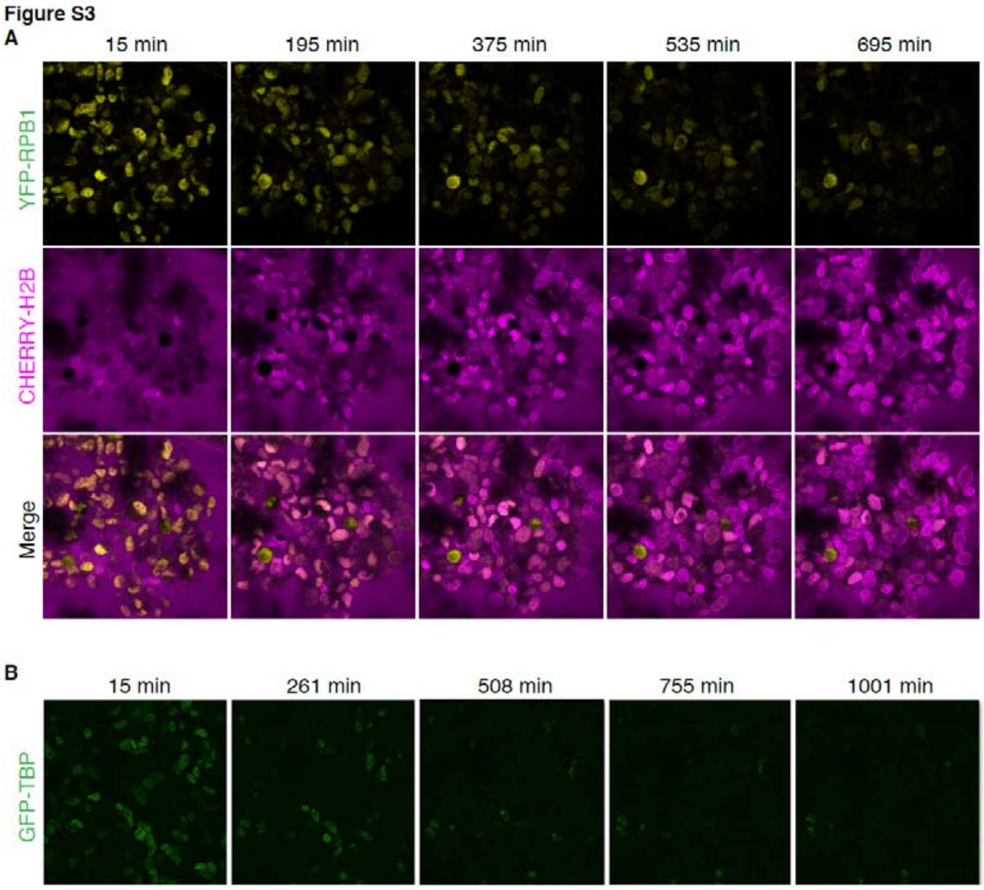

Figure S3. Somatic RNA polymerase II and TBP are lost from transplanted nuclei (related to figure 3).

(A) Somatic RPB1 is lost after nuclear transfer. Still images of confocal time-lapse imaging of YFP-RPB1 U2OS nuclei transplanted into oocytes expressing Cherry-H2B from Movie S1. Confocal images were recorded every 20 minutes for 12 hours, starting 15 minutes after nuclear transfer. YFP-RPB1 is shown in green and Cherry-H2B in magenta. Images are projected Z-sections.

(B) Confocal time-lapse imaging of GFP-TBP U2OS nuclei transplanted into oocytes. Still images of Movie S2 are shown. Confocal images were recorded every 3700 seconds overnight, starting 15 minutes after nuclear transfer. Images are projected Z-sections.

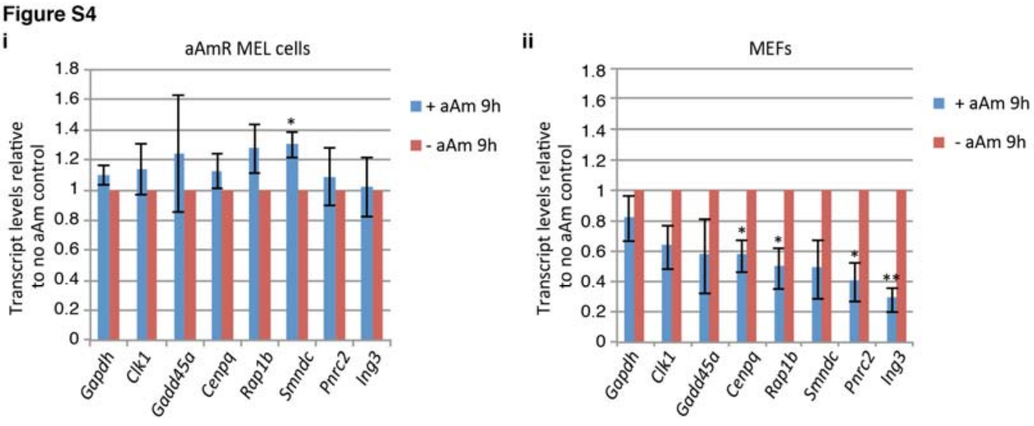

Figure S4. Erythroleukemia cells carrying alpha-Amanitin resistant RPB1 can transcribe in the presence of alpha-Amanitin (related to figure 4).

(i) Mouse erythroleukemia cells carrying alpha-Amanitin (aAm) resistant RPB1, used in Figure 4, were cultured with or without aAm (1.5 μg/ml) for 9 hr. After the treatment, the same number of cells were collected and subjected to RT-qPCR analysis. (ii) As a control of aAm-non-resistant cells, the same procedure was applied to mouse embryonic fibroblasts used in Figure 1. Data are represented as mean +/- SEM. n = 3. p values were calculated using ANOVA; *$p < 0.05$, **$p < 0.01$.

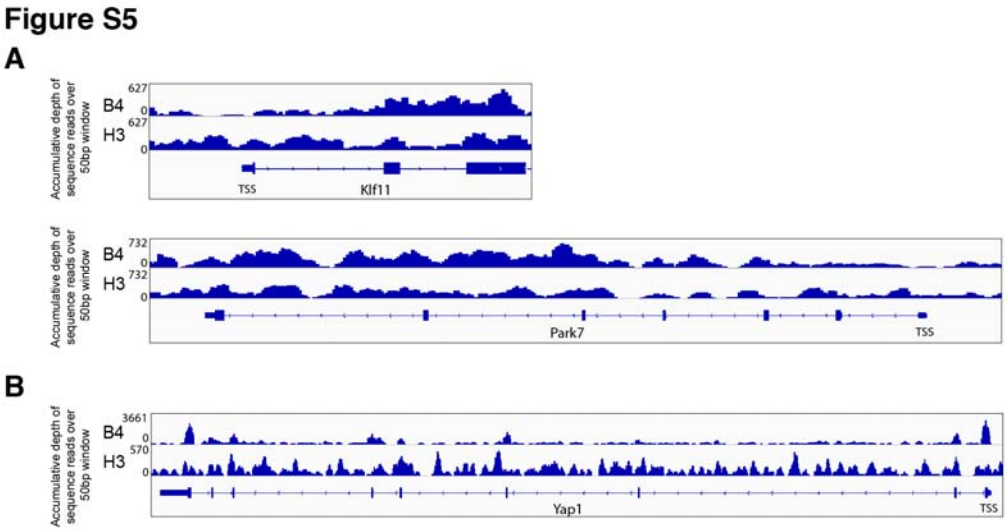

Figure S5. Genome browser tracks of histone B4 and H3 ChIP-seq data (related to figure 5).

B4 and H3 ChIP-seq tracks for **(A)** *Klf11* and *Park7*, which represent B4 depletion around TSS, and **(B)** *Yap1*, which shows B4 enrichment in exons. Exons are represented by small boxes.

SUPPLEMENTAL EXPERIMENTAL PROCEDURES

Immunofluorescence and Live Cell Imaging

GVs containing transplanted nuclei were dissected by batches of 5 in GV isolation buffer (Astrand et al., 2009) and fixed immediately in 4% paraformaldehyde/1x PBS overnight at 4°C. All following steps were carried out in transparent 1.5 ml eppendorf tubes. Fixed GVs were rinsed twice in PBS 0.2% Tween (PBT) and blocked in 5% Fetal Bovine Serum (FBS) 1x PBT (Blocking). Permeabilization was carried out for 3 minutes in 0.5% Triton X, 5% FBS 1x PBT, followed by 3 washes in blocking medium. Primary antibody overnight incubations were performed at 4°C. The primary antibodies used were: Mouse IgM anti-Serine5 phosphorylated RNA Polymerase II: H14 (1/200, Covance MMS-134R), Rabbit IgG anti-Serine2 phosphorylated RNA polymerase II CTD repeat (1/200, Abcam: ab5095), Mouse IgM anti-Serine2 phosphorylated RNA Polymerase II: H5 (1/200, Covance MMS-129R), Mouse IgG1 anti-Rpb1: ARNA3 (1/50, Millipore CBL221), Mouse IgG2a anti-unphosphorylated Ser2 RNA Polymerase II: 8WG16 (1/50, Abcam: ab817), Rabbit IgG anti-histone B4 (1/500) (Ohsumi et al., 1993). The secondary antibodies used (1/200, Invitrogen) were Alexa 488 goat anti-mouse IgG , Alexa 647 goat anti-mouse IgM, Alexa 594 donkey anti-rabbit IgG, Alexa 488 goat anti-rat IgG. Samples were mounted in ProLong Gold reagent with DAPI (Invitrogen) or in Vectashield with DAPI (H-1000, Vector Laboratories) and imaged as described (Miyamoto et al., 2013; Pasque et al., 2011) and usually involved projection of 10 Z-sections using ImageJ.

Preparation of oil GVs and live cell imaging were done as described previously (Jullien et al. 2010, Miyamoto et al. 2011). For the live cell observation of Pol II activation, 2.3 nl of Fab antibodies (100 µg/ml stock) (Hayashi-Takanaka et al., 2011) was injected into oil GVs. Ten minutes after the injection of Fab antibodies, Pol II dynamics was observed under confocal microscopes.

Sequencing Data Filtering

Quality filtering of sequencing data

Fastq files from Illumina sequencing and the SRA were filtered for low quality reads (<Q20) and low quality bases were trimmed from the ends of the reads (<Q20). Adaptors were removed from both pairs using cutadapt (Martin, 2011).

Separation of Xenopus laevis from Mus musculus

A background genome was constructed containing Mouse mm9 and *Xenopus laevis* 6.1 (Bowes et al., 2008). The sequencing reads were mapped to this background genome using bwa 0.6.2. Reads that mapped exclusively to a single genome were extracted with a further quality filter of mapping quality > 13 being applied using bamtools 2.1.1. This resulted in BAM files representing the reads that exclusively map to each genome and were used for further analysis.

RNA-seq Analysis

For *Xenopus laevis* the genome build 6.1 from Xenbase was used as a reference genome and the filtered fastq data was mapped with TopHat

version 2.0.6 (Trapnell et al., 2009) along with a junction file based on the assembly used in (Miyamoto et al., 2013) with 29,171 transcripts. For *Mus musculus* UCSC mm9 was used as the genome with RefSeq annotation obtained from the UCSC as the junction file. Reads were generated for each gene by summing the number of mapped reads overlapping its exons. These were converted to RPKMs by normalizing by the total read count for each sample and corresponding transcript lengths.

For the *Mus musculus* samples an unsupervised hierarchical clustering of the RPKM values was performed using hclust in R (using Euclidean distance and Ward agglomeration). To prepare the count data for differential expression analysis, a filter was applied requiring genes to have 1 count per million (CPMs) in all three treatment replicates or all three controls. This reduced the initial pool of 28,240 genes to 4,210 genes. Differentially expressed genes were then identified using edgeR (Robinson et al., 2010). Gene Ontology (GO) terms showing over-representation of genes that are up or down-regulated after nuclear transfer were detected using a hypergeometric test. Published RNAseq data used include GSM1033649 (mouse ESCs), GSM970539 (mouse ESCs), GSM1080200, GSM1080201 and GSM1080202 (mouse 2-cell), (Banaszynski et al., 2013; Jia et al., 2012; Xue et al., 2013).

In order to compare *Xenopus laevis* RPKM values to *Mus musculus* values ORFs were predicted from the transcripts using the Trinity suite (Grabherr et al., 2011). Orthologs were predicted using Inparanoid 4 on the predicted protein sequences (Alexeyenko et al., 2006). Two boxplots of the log(1+RPKM) values for the NT oocytes in *Mus musculus* were generated.

The plots show values where there is a *Xenopus laevis* ortholog with low expression (log(1+RPKM) < 6) and high expression (log(1+RPKM) >= 6), respectively.

ChIP-seq Analysis

ChIP-Seq analysis was performed on the genome split bam files with *Mus musculus* annotation. Peaks were called using SICER (parameterisation: redundancy threshold = 1, window size = 200, fragment size = 350, effective genome fraction = 0.74, gap size = 400, FDR = 0.05) for B4 using the H3 pulldown as control. Using the B4 and H3 pulldowns and annotation previously described, the distance to each TSS of the midpoint of all reads within 20 kb of that TSS was measured. The distances were pooled over all TSS locations and plotted in a histogram with 200 bp bins. This process was repeated for the TSS of expressed genes and non-expressed genes in transplanted nuclei and the resulting histograms were compared. The midpoints of peak locations were classified into regions of the genome including exonic, intronic, intergenic and around the TSS and TTS. The peak counts in these regions were modelled by a Poisson distribution assuming, under the null hypothesis, that the incidence rate in each was equal to that of the whole genome average. The probability of the observed counts, given this distribution, was calculated for each region to ascertain whether peak rates were significantly different to the whole genomic background.

Supplementary material – Fast track reprogramming by oocytes.

Primer list for qPCR analysis

Primer name	Sequence (5'-3')	Amplicon length (bp)	Analysis
mouse Oct4 F	GAAGGGCAAAAGATCAAGTATTGAG	78	qRT-PCR
mouse Oct4 R	GCCCCCCCTGGGAAAG		qRT-PCR
mouse Sox2 F	TCAGGCTGCCGAGAATCC	97	qRT-PCR
mouse Sox2 R	TCAAACTGTGCATAATGGAGTAAAAAC		qRT-PCR
mouse Utf1 F	ACCAGCTGTCGACCCTGAAC	63	qRT-PCR
mouse Utf1 R	AAACGGTTTGGTCGAAGGAA		qRT-PCR
mouse c-myc F	GCCCCCAAGGTAGTGATCCT	65	qRT-PCR
mouse c-myc R	TGCTCGTCTGCTTGAATGGA		qRT-PCR
mosue Mycn F	AAGTTCGGGACACTAAGGAGCTT	63	qRT-PCR
mosue Mycn R	GGAATCTTGGACCGGAACAA		qRT-PCR
mosue Gadd45a F	CGGGAAAGTCGCTACATGGA	59	qRT-PCR
mosue Gadd45a R	TCACCGTTCCGGGAGATTAA		qRT-PCR
mouse Lefty1 F	TGTGTGTGCTCTTTGCTTCC	180	qRT-PCR
mouse Lefty1 R	GGGGATTCTGTCCTTGGTTT		qRT-PCR
mouse Peg12 F	GCTGAGGGATGAGCACACTGT	59	qRT-PCR
mouse Peg12 R	TGGCAGGAGAGCCCTTAAAG		qRT-PCR
mouse Slc18a3 F	TGCAGGATGGCCTTGGA	60	qRT-PCR
mouse Slc18a3 R	CCCCACAGAAAGTGAAGATGTG		qRT-PCR
mouse Pex26 F	CCTCTTCCAGTGCCGTAAGC	56	qRT-PCR
mouse Pex26 R	TGCCAGCCCGACTCAAA		qRT-PCR
mouse Ssr1 F	TGTGTGGGTGTGGATGTCTGT	68	qRT-PCR
mouse Ssr1 R	GGTGTGTTTCAGGTAGTTTAACATTGA		qRT-PCR
mouse Clk1 F	AGGATGCCGAACATGAGCTT	62	qRT-PCR
mouse Clk1 R	GGCGGGATCATACTCCAACA		qRT-PCR
mouse Cenpq F	CCTTGAACTCCCAGCAATCC	59	qRT-PCR
mouse Cenpq R	GCTCGCCCCGGTAATCC		qRT-PCR
mouse Rap1b F	CCAACAGGGAGCCACAGTATTT	62	qRT-PCR
mouse Rap1b R	GGCCACCTCAAAGTCGTTGT		qRT-PCR
mouse Smndc F	TTTTCAGATGCTTTGCACTTGAC	62	qRT-PCR
mouse Smndc R	GGCGTTTTGCCTCCATGT		qRT-PCR
mouse Pnrc2 F	CACCCCACAAAGCTGCTAAAG	78	qRT-PCR
mouse Pnrc2 R	TCACAAACACAACTCCCTACTTCAA		qRT-PCR
mouse Ing3 F	CAGGACGCTAGGAGGAAAGACA	64	qRT-PCR
mouse Ing3 R	CTCTAAGTGGTGGCCTGGAGAT		qRT-PCR

SUPPLEMENTAL INFORMATION REFERENCES

Alexeyenko, A., Tamas, I., Liu, G., and Sonnhammer, E.L.L. (2006). Automatic clustering of orthologs and inparalogs shared by multiple proteomes. Bioinformatics *22*, e9–e15.

Astrand, C., Belikov, S., and Wrange, O. (2009). Histone acetylation characterizes chromatin presetting by NF1 and Oct1 and enhances glucocorticoid receptor binding to the MMTV promoter. Exp Cell Res *315*, 2604–2615.

Banaszynski, L.A., Wen, D., Dewell, S., Whitcomb, S.J., Lin, M., Diaz, N., Elsässer, S.J., Chapgier, A., Goldberg, A.D., Canaani, E., et al. (2013). Hira-dependent histone H3.3 deposition facilitates PRC2 recruitment at developmental loci in ES cells. Cell *155*, 107-120

Bowes, J.B., Snyder, K.A., Segerdell, E., Gibb, R., Jarabek, C., Noumen, E., Pollet, N., and Vize, P.D. (2008). Xenbase: a Xenopus biology and genomics resource. Nucleic Acids Res *36*, D761–D767.

Grabherr, M.G., Haas, B.J., Yassour, M., Levin, J.Z., Thompson, D.A., Amit, I., Adiconis, X., Fan, L., Raychowdhury, R., Zeng, Q., et al. (2011). Full-length transcriptome assembly from RNA-Seq data without a reference genome. Nat Biotechnol *29*, 644–652.

Hayashi-Takanaka, Y., Yamagata, K., Wakayama, T., Stasevich, T.J., Kainuma, T., Tsurimoto, T., Tachibana, M., Shinkai, Y., Kurumizaka, H., Nozaki, N., et al. (2011). Tracking epigenetic histone modifications in single cells using Fab-based live endogenous modification labeling. Nucleic Acids Res *39*, 6475–6488.

Jia, J., Zheng, X., Hu, G., Cui, K., Zhang, J., Zhang, A., Jiang, H., Lu, B., Yates, J. 3rd, Liu, C., et al. (2012). Regulation of pluripotency and self-renewal of ESCs through epigenetic-threshold modulation and mRNA pruning. Cell *151*, 576-589.

Jullien, J., Astrand, C., Halley-Stott, R.P., Garrett, N., and Gurdon, J.B. (2010). Characterization of somatic cell nuclear reprogramming by oocytes in which a linker histone is required for pluripotency gene reactivation. Proceedings of the National Academy of Sciences *107*, 5483–5488.

Martin, M. (2011). Cutadapt removes adapter sequences from high-throughput sequencing reads. EMBnet. Journal *17*, pp.10–pp.12.

Miyamoto, K., Pasque, V., Jullien, J., and Gurdon, J.B. (2011). Nuclear actin polymerization is required for transcriptional reprogramming of Oct4 by oocytes. Genes Dev *25*, 946–958.

Miyamoto, K., Teperek, M., Yusa, K., Allen, G.E., Bradshaw, C.R., and Gurdon, J.B. (2013). Nuclear Wave1 Is Required for Reprogramming Transcription in Oocytes and for Normal Development. Science *341*, 1002–

1005.

Ohsumi, K., Katagiri, C., and Kishimoto, T. (1993). Chromosome condensation in Xenopus mitotic extracts without histone H1. Science *262*, 2033–2035.

Pasque, V., Gillich, A., Garrett, N., and Gurdon, J.B. (2011). Histone variant macroH2A confers resistance to nuclear reprogramming. Embo J *30*, 2373–2387.

Robinson, M.D., McCarthy, D.J., and Smyth, G.K. (2010). edgeR: a Bioconductor package for differential expression analysis of digital gene expression data. Bioinformatics *26*, 139–140.

Trapnell, C., Pachter, L., and Salzberg, S.L. (2009). TopHat: discovering splice junctions with RNA-Seq. Bioinformatics *25*, 1105–1111.

Xue, Z., Huang, K., Cai, C., Cai, L., Jiang, C.Y., Feng, Y., Liu, Z., Zeng, Q., Cheng, L., Sun, Y.E., et al. (2013). Genetic programs in human and mouse early embryos revealed by single-cell RNA sequencing. Nature *500*, 593-597.
